# Circulating mortalin in blood and activation of the alternative complement pathway as risk indicators in COVID-19 infection

**DOI:** 10.3389/fimmu.2024.1337215

**Published:** 2024-04-23

**Authors:** Maya Avraham, György Sinkovits, Lisa Hurler, Zoltán Prohászka, Zvi Fishelson

**Affiliations:** ^1^ Department of Cell and Developmental Biology, The Faculty of Medical and Health Sciences, Tel Aviv University, Tel Aviv, Israel; ^2^ Department of Internal Medicine and Hematology and Research Group for Immunology and Hematology, Semmelweis University - Eötvös Loránd Research Network (Office for Supported Research Groups), Budapest, Hungary

**Keywords:** COVID-19, mortalin, Grp75, complement, overall survival, risk factor

## Abstract

**Background:**

Mortalin/GRP75 is a ubiquitous mitochondrial chaperone related to the cytosolic heat shock protein 70. It protects cells from various types of damages and from senescence. Our goal was to determine whether COVID-19 patients have circulating mortalin in their blood and to assess its prognostic value in anticipating disease severity.

**Methods:**

Mortalin was determined by ELISA in the sera of 83 COVID-19 patients enrolled in the study. Patients were categorized into 4 groups: critical patients who died (FATAL) or required intensive care and survived (ICU), patients of mild severity (hospitalized but not critical) who required nasal oxygen support (HOSP+O_2_), and patients who did not need oxygen therapy (HOSP).

**Results:**

The mortalin concentration in the serum of all COVID-19 patients in the cohort was 194-2324 pg/mL. A comparison of the mortalin levels by peak severity among the various patient groups showed a highly significant difference between the HOSP and FATAL groups and a significant difference between the HOSP and the ICU groups. COVID-19 patients who eventually failed to survive had at hospitalization a markedly higher level of mortalin in their sera. Cox regression analysis revealed a high mortality hazard (HR=3.96, p<0.01) in patients with high mortalin circulating levels (above the median, ≥651 pg/mL). This was confirmed in survival curve analysis (Kaplan-Meier; p=0.0032, log-rank test). Mortalin remained an independent predictor of mortality even after adjusting for age and sex or various complement activation products. Complement activation data collected in an earlier study in the same cohort was compared regarding the mortalin levels. Patients with higher circulating mortalin levels also had higher levels of complement C3a but reduced levels of properdin.

**Discussion:**

This is the first report on circulating mortalin in COVID-19 patients. Higher mortalin levels were associated with more severe illnesses and a higher risk of death. We claim that quantifying the blood levels of mortalin and activated complement proteins will provide important information on the prognosis of COVID-19 patients and will serve as a useful tool for guiding their clinical management and treatment.

## Introduction

The COVID-19 pandemic started in December 2019 and rapidly spread throughout the world. As updated by the WHO in October 2023, the number of confirmed cases infected by the SARS-CoV-2 virus is higher than 770 million and the number of confirmed deaths is close to 7 million (1). Some of the infected patients develop a more severe illness, and experience an extensive systemic inflammatory response with a “cytokine storm”, severe lung damage, acute respiratory distress syndrome (ARDS), multiorgan failure, and a higher risk of death (2). This has been attributed to several risk factors, including age and medical conditions such as cardiovascular disease, diabetes, chronic respiratory disease, or cancer (3–5). Supportive life-saving treatments have been proposed and are being applied to severely sick COVID-19 patients with an understanding that patients who need them should receive them as early as possible (6). Hence, identifying early indicators of severe disease and mortality is essential.

To date, several markers have been proposed for early detection of severe COVID-19. SARS-CoV-2 virus activates the complement system of the patients, leading to the accumulation of cleaved fragments of complement C3 and C5, C3a, and C5a in the patients’ blood (7–9). Apparently, C3a and C5a play a significant role in triggering the cytokine storm and ARDS in the patients (8, 10–12). This has led to clinical trials targeting, for example, complement C5 with Ravulizumab and testing its effect on COVID-19-associated lung injuries and distress (7, 13). Moreover, complement activation as well as increased levels of C3a and C5a were found to be associated with advanced disease severity (8, 14). Soluble complement C5b-9 complex (sC5b-9) was also identified in the plasma of COVID-19 patients and was higher in patients with severe disease than in patients with moderate disease (15). It was proposed that sC5b-9 may be a more stable and reliable indicator of *in-vivo* complement activation than C5a (15).

Massive cellular damage occurs in the body after injury or during viral infection. This leads to the release of damage-associated molecular patterns (DAMPs), which are known to activate a systemic inflammatory response (16). Extensive cell damage may also affect the mitochondria, leading to the release of mitochondrial DAMPs, such as mitochondrial DNA (mtDNA) into the blood (17). Thus, higher levels of circulating mtDNA were measured in the blood of COVID-19 patients who eventually died or required ICU admission (18). High circulating mtDNA was apparently an indicator of poor COVID-19 outcomes.

Mortalin, also known as the mitochondrial stress protein 70 and GRP75 (gene: Hspa9), is an indispensable mitochondrial matrix protein. It is essential for the transport of proteins into mitochondria and has other important functions that support cell stress, survival, and aging (19). Once mortalin activity is blocked, cells fail to survive (20, 21). We previously reported that colorectal carcinoma patients have circulating mortalin in their blood plasma and that higher mortalin levels indicate a poor prognosis in terms of survival (22, 23). In addition, we have shown that mortalin can be released in extracellular vesicles from complement-activated cells (24). Identification of complement activation markers (increased levels of circulating C3a, C5a, and sC5b-9) as well as cellular mitochondrial damage indicators (circulating mtDNA) in COVID-19 patients, as described above, prompted us to look for circulating mortalin in the blood of COVID-19 patients. As reported here, variable levels of mortalin were found in COVID-19 patients’ blood. Interestingly, patients with higher mortalin levels experienced a more severe illness and had a shorter survival.

## Methods

### Patients, clinical categories, and serum samples

This study included a cohort of adult COVID-19 patients (83 individuals confirmed positive by PCR) who were admitted to two tertiary hospitals in Budapest between April 20 and July 2, 2020. Hence, this cohort represents the COVID-19 pathophysiology before the vaccine era, with wild-type SARS-CoV-2 infection. Additionally, 7 blood donors post-COVID-19 infection not requiring hospitalization served as the control group. All participants were part of a larger COVID-19 cohort described in an earlier report (14); however, the serum samples for the mortalin analysis were only available for the individuals described here. The severity of patients’ morbidity was categorized as described previously (14). In brief, critical patients were those who died (FATAL) or required intensive care and survived (ICU). Patients with milder severity (hospitalized but not critical) and that required nasal oxygen support constituted the HOSP+O_2_ group, whereas patients who did not need oxygen therapy constituted the HOSP group. Respiratory failure necessitating mechanical ventilation occurred in 60% and 78.3% of the ICU and FATAL groups, respectively. Macro-thromboembolic complications occurred in 46.7% and 4.3% of the ICU and FATAL groups, respectively. Acute kidney injury (KDIGO: 2-3) occurred in 8.3%, 6.7%, and 39.1% of the HOSP+O_2_, ICU, and FATAL groups, respectively (14).

For each enrolled patient, the sample taken at hospital admission was included in the study. Laboratory data (blood count, clinical chemistry, coagulation, and inflammatory parameters) were extracted from the hospital records. Blood samples were collected from the antecubital vein or from a central venous catheter and were immediately transferred to the processing laboratory for serum separation. The EDTA plasma and serum samples were immediately frozen and stored at -70°C or on dry ice until tested.

The study was conducted in accordance with the Declaration of Helsinki and its subsequent revisions and was approved by the Hungarian Scientific and Research Ethics Committee (ETT-TUKEB; No. IV/4403–2/2020/EKU). Written informed consent was obtained from the patients and the control subjects or from the closest relative available, if the patient was unable to give informed consent.

### Mortalin ELISA

Mortalin levels in the serum samples were measured using the Mortalin ELISA kit from LSBio (Seattle, WA, USA), according to the manufacturer’s instructions. Briefly, test serum samples, diluted 1:3, in duplicate, were added to microtiter plate wells precoated with anti-mortalin antibodies (the capturing antibodies) for 1 h at 37°C. For the standard curve, wells were treated with serially diluted recombinant mortalin (5-40 pg/well) mixed with diluted (1:3) normal human serum. Wells treated with NHS (diluted 1:3) only served as negative controls and gave no signal above the Zero control provided in the kit. A biotin-conjugated anti-mortalin detection antibody was added for 1 h at room temperature. After a wash, the bound biotin was reacted with horseradish-peroxidase (HRP) conjugate, followed by washing and TMB substrate colorimetric detection at 450 nm using a microplate reader (Spectrafluor plus, Tecan, Austria). The Mortalin concentration in the test samples was calculated by using the standard curve.

### Complement analyses

The complement classical, lectin, and alternative pathway activities as well as the concentrations of complement C3, C4, C1q, factors H, I, and B, and the activation markers C3a and sC5b-9 were determined as described earlier (14). The concentrations of properdin (HK334, Hycult Biotech), C1s/C1-INH complex (HK399, Hycult Biotech), MASP-1/C1-INH complex (HK3001, Hycult Biotech), C4a (A035, Quidel), C4d (A009, Quidel), and Bb (A027, Quidel) were determined in blood plasma-EDTA samples by using commercially available ELISA kits. All assays were performed according to the manufacturer’s instructions.

### Statistical analyses

The distribution of most continuous variables was skewed; therefore, these data were presented as the median and interquartile range, and nonparametric tests were used: the Mann-Whitney test for comparing two independent groups, the Kruskal-Wallis test with Dunn’s post-test for comparing more than two independent groups, and Spearman’s rank correlation test for testing potential correlations between continuous variables. Cases with missing values were excluded pairwise. All applied tests were two-tailed, with the false discovery rate (alpha) of 0.05. The Benjamini-Hochberg procedure was used to correct the level of significance in order to maintain a false discovery rate of 5%. Uni- and multivariable Cox proportional hazard models were used to assess the effects of mortalin and various clinical and laboratory parameters on in-hospital mortality. Kaplan-Meier curves of selected subgroups were compared by the log-rank test in a pairwise manner. Statistical analyses were performed using Statistica 13.5 and GraphPad Prism 9 software.

## Results

**Figure 1:**
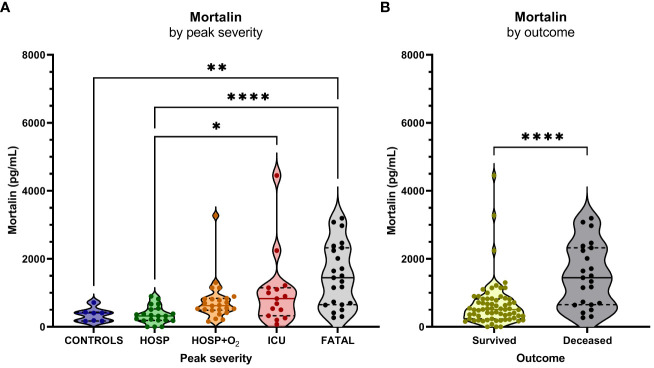
Mortalin levels and the COVID-19 disease outcome. **(A)** Mortalin levels by peak disease severity. Asterisks indicate the p-values obtained by the Kruskal-Wallis test with Dunn’s multiple comparison *post hoc* test (* p<0.05, ** p<0.01, and **** p<0.0001), non-significant differences are not indicated. **(B)** Relationship between mortalin levels and mortality. Asterisks indicate the p-value obtained by the Mann Whitney test (**** p<0.0001).

**Figure 2:**
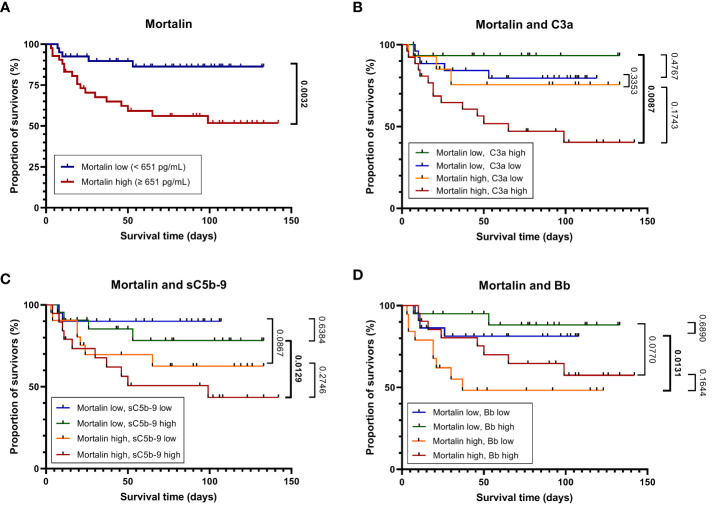
Mortality of patients with COVID-19 as stratified by high or low mortalin levels. Kaplan-Meier curves (in-hospital all-cause mortality plotted against the time from hospital admission to death or the last follow-up) for patients with mortalin levels above or below the median (651 pg/mL) are shown **(A)**. The above groups were further divided based on the levels of the complement activation markers C3a, sC5b-9, and Bb above or below the median (251 and 301 ng/mL, and 1.723 μg/mL, respectively). The Kaplan-Meier curves of these subgroups are shown in **(B–D)**, respectively. For pairwise comparisons of certain groups, the p-values of log-rank tests are shown; significant differences are denoted in bold.

The concentration of soluble mortalin was quantified in the sera of 83 hospitalized COVID-19 patients and 7 controls. In the hospitalized patients, the mortalin levels ranged from 194-2324 pg/mL, with a median value of 651 pg/mL (**Table 1**). In the control group, the mortalin concentrations varied between 172 and 433 pg/mL. Comparison of the mortalin levels among the various patient groups (**Figure 1A**) showed a highly significant difference between the HOSP (627 (495-834) pg/mL) and the FATAL (1443 (651-2324) pg/mL) groups (p<0.0001) and a significant difference between the HOSP and the ICU (830 (328-1150) pg/mL) groups (p=0.0385). Comparison of the mortalin values by the Mann-Whitney test showed markedly higher levels of mortalin in sera from COVID-19 patients who eventually failed to survive (the difference between survived (510 (236-822) pg/mL) and deceased (1443 (651-2324) pg/mL) patients was highly significant; p<0.0001; **Figure 1B**). Survival analysis of the patients (Kaplan-Meier) divided into high mortalin (≥651 pg/mL median level) and low mortalin (<651 pg/mL) groups showed a significantly shorter survival (log-rank overall comparison of p=0.0032) of patients with high mortalin levels (**Figure 2A**).

**Table 1:** Mortalin and Laboratory data of the subclasses of patients.

Variables	Total hospitalized, n=83	Hospitalized, no oxygen support, n=21 (HOSP)	Hospitalized, with nasal oxygen support, n=24 (HOSP+O_2_)	ICU, n=15	Fatal, n=23	Control, n=7	p-value*
Age (median, IQR)	70 (57-78)	56 (42-69)	72 (64-80)	59 (54-68)	76 (72-83)	31 (30-42)	**<0.0001**
Male sex, % (n)	56.6 (47)	66.7 (14)	58.3 (14)	53.3 (8)	47.8 (11)	0.0 (0)	0.6410
Delay between first symptoms and blood sampling, days (median, IQR)	9 (4-17)	10 (5-14)	8 (5-16)	9 (6-28)	6 (2-17)	62 (40-72)	0.5343
Comorbidities, % (n)							
Hypertension	65.1 (54)	47.6 (10)	66.7 (16)	66.7 (10)	78.3 (18)	14.2 (1)	0.2020
Diabetes mellitus	25.3 (21)	14.3 (3)	29.2 (7)	13.3 (2)	39.1 (9)	0.0 (0)	0.1716
Chronic pulmonary disease	21.7 (18)	9.5 (2)	16.7 (4)	26.7 (4)	34.8 (8)	0.0 (0)	0.1929
Chronic heart disease	33.7 (28)	23.8 (5)	45.8 (11)	13.3 (2)	43.5 (10)	0.0 (0)	0.0993
Malignant disease	25.3 (21)	14.3 (3)	8.3 (2)	46.7 (7)	39.1 (9)	0.0 (0)	**0.0143**
Laboratory findings (median, IQR)**							
**Mortalin (pg/mL)**	**651** (345-1150)	**321** (194-510)	**627** (495-834)	**830** (328-1150)	**1443** (651-2324)	**405** (172-433)	**<0.0001**
White blood cell count (G/L)	6.7 (4.8-8.7)	5.6 (4.8-7.4)	7.0 (4.9-8.4)	6.6 (4.1-7.8)	7.5 (4.6-11.6)	7.1 (5.6-7.5)	0.4610
Neutrophil granulocyte count (G/L)	5.0 (2.9-6.7)	3.7 (2.5-4.9)	4.6 (2.9-6.1)	5.0 (3.2-6.4)	6.0 (3.2-10.3)	4.0 (2.9-4.6)	0.0297
Monocyte count (G/L)	0.42 (0.27-0.58)	0.44 (0.30-0.72)	0.44 (0.32-0.56)	0.44 (0.21-0.57)	0.42 (0.18-0.52)	0.51 (0.32-0.52)	0.6960
Lymphocyte count (G/L)	1.0 (0.8-1.6)	1.5 (1.0-2.0)	1.3 (0.9-1.8)	0.9 (0.7-1.1)	0.9 (0.5-1.2)	2.2 (2.0-2.4)	**0.0011**
Red blood cell count (T/L)	4.10 (3.51-4.64)	4.47 (3.80-4.80)	4.19 (3.76-4.61)	3.68 (3.18-4.43)	3.87 (3.22-4.56)	4.37 (4.23-4.47)	0.0800
RDW (CV%)	14.7 (13.1-16.0)	13.2 (12.7-15.0)	13.9 (13.0-16.3)	14.6 (13.6-15.6)	15.8 (14.7-17.4)	13.1 (12.2-14.1)	**0.0003**
Platelet count (G/L)	227 (165-291)	236 (190-279)	231 (170-368)	229 (147-257)	196 (129-291)	266 (216-276)	0.5227
INR	1.09 (0.99-1.20)	1.02 (0.99-1.11)	1.02 (0.98-1.12)	1.12 (1.02-1.17)	1.17 (1.07-1.48)	–	**0.0067**
D-Dimers (ng/mL)	1403 (841-2613)	1480 (610-2990)	941 (625-1515)	1798 (879-3090)	1430 (1100-4380)	212 (174-253)	0.0639
Fibrinogen (g/L)	5.3 (4.1-6.5)	4.9 (4.1-5.3)	5.0 (4.4-6.4)	6.9 (5.4-7.9)	5.0 (3.9-6.5)	–	0.1853
LDH (U/L)	299 (215-596)	188 (166-239)	279 (204-343)	617 (357-850)	608 (370-1041)	–	**<0.0001**
AST (U/L)	35 (21-52)	22 (17-32)	29 (18-44)	46 (28-65)	52 (32-82)	–	**0.0003**
ALT (U/L)	26 (14-46)	17 (13-24)	27 (13-38)	35 (26-67)	45 (14-77)	–	**0.0096**
GGT (U/L)	53 (25-105)	33 (23-72)	59 (23-76)	117 (25-155)	72 (26-91)	–	0.2991
ALP (U/L)	79 (61-129)	71 (56-87)	71 (56-85)	105 (61-165)	106 (73-146)	–	0.0527
Bilirubin (μmol/L)	9.1 (6.8-11.1)	8.4 (6.2-10.4)	9.5 (7.7-13.2)	7.9 (6.8-10.2)	9.7 (6.6-11.9)	–	0.5446
Troponin (ng/mL)	26.5 (8.0-51.0)	12.5 (5.5-25.5)	19.0 (4.0-33.0)	13.0 (3.0-28.0)	51.0 (32.0-91.5)	–	**0.0049**
BUN (mmol/L)	6.4 (4.6-13.3)	5.5 (3.9-6.5)	5.3 (4.1-6.8)	7.5 (3.8-13.5)	14.4 (7.5-17.6)	–	0.0002
Creatinine (μmol/L)	78 (59-125)	73 (50-92)	74 (61-91)	92 (54-116)	144 (62-205)	–	0.0863
Protein (g/L)	61 (55-64)	65 (63-70)	61 (55-64)	57 (54-63)	55 (50-61)	74 (71-79)	**0.0006**
Albumin (g/L)	32 (27-38)	35 (30-37)	33 (27-38)	30 (26-37)	29 (25-34)	48 (46-51)	0.1117
Neu/Ly	5.2 (2.1-8.0)	1.8 (1.1-4.1)	4.4 (2.4-5.7)	5.8 (4.5-8.9)	9.8 (5.8-14.2)	1.8 (1.5-2.1)	**<0.0001**
CRP (mg/L)	69.4 (16.2-154.6)	14.6 (8.2-61.9)	35.4 (15.2-95.3)	116.3 (93.2-195.4)	149.1 (39.6-196.8)	1.0 (0.7-2.5)	**<0.0001**
PCT (ng/mL)	0.11 (0.03-0.30)	0.03 (0.02-0.06)	0.06 (0.02-0.19)	0.21 (0.13-1.42)	0.34 (0.11-1.21)	–	**<0.0001**
IL-6 (pg/mL)	34.1 (12.6-90.4)	12.6 (6.0-25.0)	30.0 (9.7-65.5)	40.6 (15.0-68.5)	148.1 (34.6-592.9)	7.2 (7.2-7.2)	**0.0002**
Ferritin (ng/mL)	622 (310-1321)	348 (199-630)	393 (199-747)	1342 (1027-2358)	932 (423-2111)	–	**<0.0001**
Haptoglobin (g/L)	2.89 (1.83-3.86)	2.81 (1.92-3.50)	2.82 (1.96-3.60)	3.05 (1.76-4.18)	3.03 (1.36-3.91)	1.09 (0.99-1.78)	0.9896

Mortalin levels measured in the sera of COVID-19 patients were compared according to the peak severity. *, p-values were obtained for nominal variables by the chi-square test, and for continuous variables by the Kruskal-Wallis test. Only the severity subgroups of hospitalized patients were compared by the above statistical tests. Results of control patients are shown for reference only; this group was not included in the statistical analyses. Missing data were not involved in calculating the percentages. Significant differences appear in bold (p<0.0259, the limit was obtained after 5% false discovery rate correction using the Benjamini-Hochberg method for comparisons of laboratory parameters). **, IQR, interquartile range; RDW, red blood cell distribution width; INR, blood coagulation international normalized ratio; LDH, lactate dehydrogenase; AST, aspartate transaminase; ALT, alanine aminotransferase; GGT, gamma-glutamyltransferase; ALP, alkaline phosphatase; BUN, blood urea nitrogen; CRP, C-reactive protein; PCT, procalcitonin; IL-6, interleukin 6; NA, not applicable/not available.

**Figure 3:**
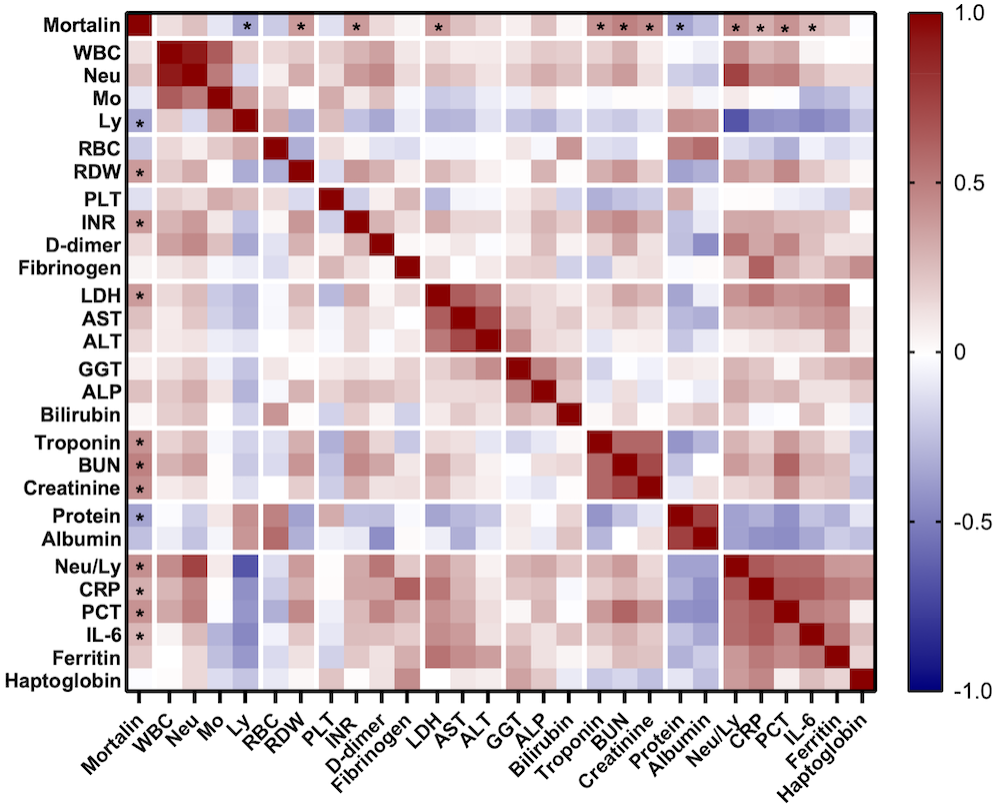
Correlation between mortalin levels and several routine laboratory parameters. Color-coding indicates the strength of each correlation (Spearman correlation coefficients); asterisks indicate significant correlations of mortalin levels (* p<0.018, the limit obtained after 5% false discovery rate correction using the Benjamini-Hochberg method for all correlations). WBC, white blood cell count; Neu, neutrophil granulocyte count; Mo, monocyte count; Ly, lymphocyte count; RBC, red blood cell count; RDW, red blood cell distribution width; PLT, platelet count; INR, international normalized ratio; LDH, lactate dehydrogenase; AST, aspartate transaminase; ALT, alanine aminotransferase; GGT, gamma-glutamyltransferase; ALP, alkaline phosphatase; BUN, blood urea nitrogen; CRP, C-reactive protein; PCT, procalcitonin; IL-6, interleukin 6.

More comorbidity and laboratory data of this cohort of patients is also shown in **Table 1**. Age of the patients and co-existence of a malignant disease significantly correlated with COVID-19 disease severity. Samples were taken at hospital admission, 9 (4-17) days after the appearance of the first symptoms. The time from disease onset until sampling did not differ in groups of hospitalized patients with different COVID-19 severity. Of the multiple laboratory tests performed, the parameters that were found to significantly reflect the severity of the disease were as follows: lymphocyte counts (Ly), RDW, INR, LDH, AST, ALT, troponin, protein, the neutrophil to lymphocyte ratio (Neu/Ly), CRP, PCT, IL-6, and ferritin. Consequently, those laboratory parameters were compared between patients who had higher mortalin levels (≥651 pg/mL) and patients with lower mortalin levels (**Table 2**). Interestingly, patients with higher mortalin levels in their blood also had higher Neu/Ly, RDW, INR, BUN, and PCT, and lower Ly levels in their blood. Their LDH, troponin, creatinine, CRP and ferritin levels also tended to be higher (with 0.013<p<0.1) in the high mortalin level patients (**Table 2**). Correlation analysis of the mortalin levels and the laboratory parameters confirmed that the levels of Neu/Ly, RDW, INR, LDH, troponin, BUN, Creatinine, CRP, PCT, and IL-6 indeed directly correlated with the mortalin levels (**Figure 3**).

**Table 2:** Laboratory and complement data of cases with mortalin concentrations below or above the median.

Variables	Mortalin Low (<651 pg/mL) n=41	Mortalin High (≥651 pg/mL) n=42	p-value*
White blood cell count (G/L)	6.3 (4.6-7.8)	6.9 (5.1-10.8)	0.3071
Neutrophil granulocyte count (G/L)	3.8 (2.8-6.0)	5.4 (3.0-9.1)	0.0711
Lymphocyte count (G/L)	1.1 (0.9-1.8)	0.9 (0.6-1.4)	**0.0075**
Monocyte count (G/L)	0.44 (0.32-0.58)	0.42 (0.25-0.55)	0.3027
Neu/Ly	3.2 (1.5-5.4)	5.9 (2.8-10.5)	**0.0059**
Red blood cell count (T/L)	4.33 (3.84-4.68)	3.92 (3.22-4.56)	0.0298
RDW (CV%)	13.8 (13.0-14.8)	15.4 (13.9-17.2)	**0.0003**
Platelet count (G/L)	236 (182-299)	226 (163-268)	0.3435
INR	1.02 (0.98-1.10)	1.16 (1.06-1.46)	**0.0023**
D-Dimers (ng/mL)	1300 (610-2980)	1430 (995-2245)	0.3704
Fibrinogen (g/L)	5.0 (4.1-6.3)	5.5 (4.0-7.4)	0.6618
LDH (U/L)	283 (199-468)	379 (229-787)	0.0305
AST (U/L)	31 (22-50)	40 (20-53)	0.4977
ALT (U/L)	26 (15-38)	28 (14-52)	0.5696
GGT (U/L)	39 (25-97)	71 (25-119)	0.1837
ALP (U/L)	71 (58-84)	106 (68-146)	0.0155
Bilirubin (μmol/L)	9.7 (7.5-11.0)	8.4 (6.6-11.1)	0.7728
Troponin (ng/mL)	15.0 (5.0-35.0)	33.0 (12.0-74.0)	0.0359
BUN (mmol/L)	5.4 (3.9-7.7)	9.5 (5.3-16.4)	**0.0021**
Creatinine (μmol/L)	74 (53-92)	107 (66-180)	0.0181
Protein (g/L)	64 (57-70)	61 (52-63)	0.1323
Albumin (g/L)	35 (28-43)	32 (27-35)	0.4889
C-reactive protein (mg/L)	53.6 (12.0-104.7)	86.8 (24.7-169.1)	0.0628
PCT (ng/mL)	0.07 (0.02-0.18)	0.17 (0.05-0.95)	**0.0112**
IL-6 (pg/mL)	25.0 (9.5-63.8)	39.2 (14.3-188.9)	0.1413
Ferritin (ng/mL)	511 (259-988)	784 (414-1780)	0.0765
Haptoglobin (g/L)	2.62 (1.69-3.46)	2.74 (1.54-4.06)	0.9170
Complement parameters			
Classical pathway activity (CH50/mL)	74 (61-89)	71 (48-81)	0.0794
Lectin pathway activity (35-125%)	59 (1-134)	56 (15-120)	0.9382
Alternative pathway activity (70-125%)	91 (78-106)	84 (60-97)	0.0771
C3 (0.9-1.8 g/L)	1.3 (1.1-1.4)	1.2 (0.9-1.3)	0.0684
C4 (0.15-0.55 g/L)	0.38 (0.30-0.47)	0.31 (0.23-0.53)	0.2822
C1q (60-180 mg/L)	108 (87-128)	107 (91-145)	0.2567
Factor H (250-880 mg/L)	734 (536-879)	681 (440-1019)	0.7952
Factor I (60-130%)	105 (90-119)	96 (78-119)	0.1270
Factor B (60-130%)	120 (100-148)	116 (91-138)	0.1703
Properdin (ng/mL)	14505 (12265-18243)	12680 (9335-15432)	0.0307
sC5b-9 (110-252 ng/mL)	308 (193-429)	287 (221-483)	0.8095
C3a (70-270 ng/mL)	217 (141-350)	333 (174-444)	0.0231
Bb (μg/mL)	1.71 (1.28-2.26)	1.99 (1.18-2.75)	0.3987
C4a (ng/mL)	2542 (1562-3818)	2122 (1338-3597)	0.5811
C4d (μg/mL)	3.99 (2.97-5.87)	3.59 (2.21-6.11)	0.5466
C1s/C1-INH (786-2906 ng/mL)	2347 (1919-2943)	2536 (2076-3399)	0.2590
MASP-1/C1-INH (13.2 - 87.9 ng/mL)	51.7 (34.5-67.9)	52.8 (37.7-75.0)	0.9473

*p-values of the Mann-Whitney U test are shown; the p-values of significant differences appear in bold (p<0.0130, the limit obtained after 5% false discovery rate correction using the Benjamini-Hochberg method for comparing all laboratory and complement parameters). For complement parameters, reference ranges are indicated in brackets, where available. RDW, red blood cell distribution width; INR, international normalized ratio; LDH, lactate dehydrogenase; AST, aspartate transaminase; ALT, alanine aminotransferase; GGT, gamma-glutamyltransferase; ALP, alkaline phosphatase; BUN, blood urea nitrogen; CRP, C-reactive protein; PCT, procalcitonin; IL-6, interleukin 6. The p-values of significant differences appear in bold.

**Figure 4:**
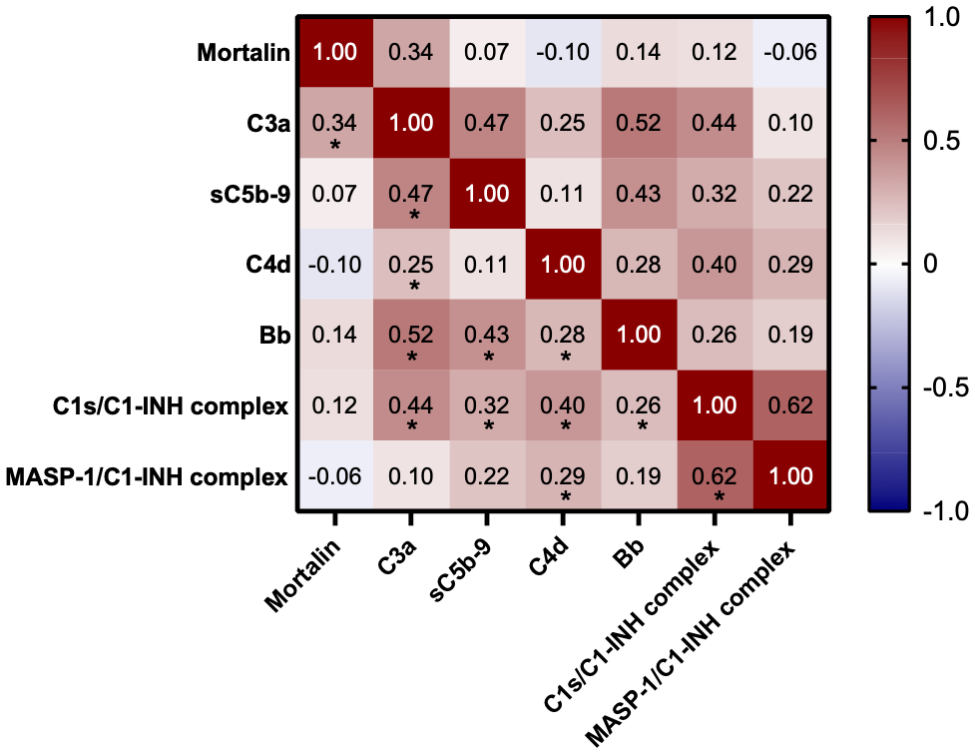
Correlation between mortalin levels and complement parameters. Color-coding indicates the strength of each correlation (Spearman correlation coefficients); asterisks indicate significance (* p<0.03, the limit obtained after 5% false discovery rate correction using the Benjamini-Hochberg method).

Measurements of the complement pathway parameters were also available for these patients from an earlier study (14); they were also compared with patients having higher and lower mortalin levels. As shown in **Table 2**, of the various parameters tested, only reduced properdin levels and higher C3a levels (p<0.05) were associated with higher mortalin levels. Accordingly, correlation analysis revealed a significant correlation between mortalin and C3a levels (**Figure 4**). A positive correlation, yet statistically insignificant, perhaps due to the size of the cohort, was also seen between higher mortalin and higher sC5b-9 or Bb levels.

Univariate analysis of the risk of mortality by Cox regression of mortalin concentration is presented in **Table 3**. High mortalin levels (above the median) significantly (p=0.0026) increased the hazard ratio to 3.96 (95% CI 1.47-10.68), when compared to low mortalin levels (below the median). Multivariable analysis of the risk of mortality was also performed, where high/low mortalin levels were adjusted for age and sex. In the adjusted model, mortalin showed a significant age- and sex-independent association with mortality (HR 3.07, CI 1.13-8.33, **Table 3**). Multivariable analysis, combining mortalin levels with complement C3a, sC5b-9, or Bb levels, was also performed (**Table 3**). These analyses revealed that the HR of higher mortalin levels remained high (3.07, 3.58, and 4.02, correspondingly) even when combined with C3a, sC5b-9, or Bb levels, thus demonstrating their independence in predicting the disease severity.

**Table 3:** Associations between mortalin levels and in-hospital mortality.

3A: Univariable	HR	HR (95% CI)	p-value
**Mortalin (**≥**651 pg/mL)**	3.96	1.47-10.68	**0.0026**
3B: Multivariable (adjusted)	HR	HR (95% CI)	p-value
**Mortalin (**≥**651 pg/mL)**	3.07	1.13-8.33	**0.0281**
**Age (year)**	1.06	1.02-1.11	**0.0047**
**Sex (male)**	0.79	0.34-1.83	0.5799
3C: Multivariable (C3a)	HR	HR (95% CI)	p-value
**Mortalin (**≥**651 pg/mL)**	3.58	1.29-9.89	**0.0140**
**C3a (**≥ **251 ng/mL)**	1.49	0.61-3.63	0.3847
3D: Multivariable (sC5b-9)	HR	HR (95% CI)	p-value
**Mortalin (**≥**651 pg/mL)**	4.02	1.49-10.84	**0.0060**
**sC5b-9 (**≥ **301 ng/mL)**	1.63	0.70-3.82	0.2603
3E: Multivariable (Bb)	HR	HR (95% CI)	p-value
**Mortalin (**≥**651 pg/mL)**	4.24	1.57-11.49	**0.0045**
**Bb (**≥ **1.723 μg/mL)**	0.54	0.23-1.26	0.1553

Results (hazard ratios and p-values) of Cox proportional hazards models of in-hospital mortality are shown. The p-values of significant differences appear in bold. **Table 3A** shows the results of a univariable model based on mortalin levels below or above the median value (651 pg/mL). **Table 3B** shows the results of a multivariable model adjusted to a baseline model consisting of age and sex. **Tables 3C–E** show the results of multivariable models involving mortalin levels and the levels of complement activation markers C3a (3C), sC5b-9 (3D), and Bb (3E) below or above the respective median value in the cohort.

To further examine the latter suggestion, we performed survival analyses of the patients by combining the mortalin and C3a levels, the mortalin and sC5b-9 levels, and the mortalin and Bb levels (**Figures 2B–D**). As shown in **Figure 2B**, patients who had high mortalin levels in addition to high (above the median) C3a levels had a significantly shorter survival (log-rank overall comparison of p=0.0087) relative to patients who had low mortalin and high C3a levels. Similarly, high mortalin, in addition to high sC5b-9 levels, predicted shorter patient survival (**Figure 2C**) compared with patients with equally high sC5b-9 but low mortalin levels. In contrast, high mortalin levels were associated with shorter survival in the group of low (and not high) Bb levels (**Figure 2D**). Interestingly, low Bb (rather than high) levels were associated with shorter survival (data not shown). Thus, we concluded that analysis of mortalin and markers of complement activation in COVID-19 patients’ sera has a high prognostic value and can help in identifying patients at low risk as well as high risk of mortality.

## Discussion

Our results show, for the first time, that COVID-19 patients have elevated levels of circulating mortalin in their blood (the measured concentration levels varied between 194 and 2,324 pg/mL). Most importantly, patients with higher mortalin levels appear to be at significant risk of mortality. This in contrast to the insignificant levels of soluble mortalin found in the blood of healthy individuals (23) and the measured levels of circulating mortalin in the 7 blood donors post-COVID-19 infection, who did not require hospitalization (172-433 pg/mL). Comparing the survival curves of patients with high and low mortalin levels strongly supported the prognostic strength of the level of circulating mortalin in patients’ blood. Ninety percent of patients with below-median concentrations of mortalin survived after 120 days, but only 50% of the patients in the high mortalin group survived up to this time. Extracellular circulating mortalin has been shown to have prognostic value in identifying patients at high risk of shorter survival in patients with colorectal carcinoma (22, 23). In both cases, the source for the mortalin circulating in the blood remains to be determined.

As described in the Introduction, complement activation and variable levels of C3a (8, 14) and sC5b-9 (15) were identified in COVID-19 patients’ blood, showing a direct correlation with the disease severity. After dividing the patients into 2 groups according to their mortalin level (low vs. high, based on the median value), the data did not show a significant difference in complement activation patterns between the two groups. However, the observed data tendencies led us to hypothesize that having a larger number of patients in the two groups might have shown a significantly reduced classical and alternative pathway activity, reduced C3 and properdin levels, and elevated C3a levels in the high mortalin group (**Table 2**). A reduced C3 level may in part account for reduced classical and alternative pathway activities and a reduced properdin level may further account for reduced alternative pathway activity. Similar levels of sC5b-9 are seen in the 2 groups. The interactions between the mortalin levels and the levels of the complement activation products as predictors of mortality were tested by Cox proportional hazard models and by Kaplan Meier survival plotting. The fact that mortalin remains in the Cox proportional hazard model as an independent predictor of mortality even after adjusting for each of the tested complement activation products indicates that mortalin is also associated with mortality via complement-independent mechanisms. However, when analyzing the Kaplan-Meier curves of the different subgroups, we observed that high mortalin values are significantly associated with shorter survival times only in patients with high C3a or sC5b-9 or with low Bb values. This observation indicates that the association between high mortalin levels and a higher risk of mortality is partly caused by both complement-mediated mechanisms as well as complement-independent ones. The first two associations (interaction with C3a and sC5b-9) seem to be logical, since they indicate a complement activation process, but the association with low Bb is intriguing. However, taking into account that (a) the alternative complement pathway is more activated in high mortalin level patients, (b) the properdin level is reduced in high mortalin level patients, and (c) lower Bb is associated with a more severe disease, we hypothesize that COVID-19 patients have a more severe disease following activation of the alternative complement pathway, leading to a more stable fixation of the alternative pathway C3/C5 convertases C3bBbP and C3bBbC3bP on the affected tissue. Consequently, the C3a level in blood is elevated, whereas the properdin (P) level is reduced because it is attached to the affected tissue. Deposition of more stable convertases on the affected tissue will generate higher levels of C5a and deposited C5b-9, which will indirectly and directly cause more severe damage and mortality. These findings and the hypothesis should be tested further with a larger cohort of COVID-19 patients including patients from other medical centers.

The correlation between mortalin level and the level of the laboratory markers of inflammation and tissue damage (e.g., CRP, LDH) observed in COVID-19 patients suggests that mortalin is released from dying or damaged cells. This may parallel the observed spike in circulating mtDNA in COVID-19 patients’ blood (18). However, cumulative data supports a claim that mortalin protects cells against numerous insults (19, 25). We reported that in response to complement-mediated damage, cells use mortalin to limit their damage by relocating from the mitochondria to the plasma membrane and out of the cells while exporting the complement membrane attack complex C5b-9 (26). It is conceivable that some of the extracellular mortalin observed in COVID-19 patients’ blood reflects such an attempt of their affected tissues to reduce and avoid the damage. Here, the circulating mortalin should be regarded as a stress reporter rather than a tissue damage marker. Activation of the complement system has been described in COVID-19 patients and even implicated in the tissue damage evident in severely ill patients (7–10). It is possible that some of the mortalin that is released from complement-attacked cells in the patients’ body reflects the affected tissue’s attempt to repair the lytic damage and some is released from dying cells. As the complement activation increases, more extracellular mortalin is measured in the blood of COVID-19 patients. Whether mortalin released from stressed and dying cells has the same or a different molecular form remains to be determined.

Another important point to consider is whether mortalin plays a protective role against viral entry. It was reported that mortalin reduces the entry of porcine epidemic diarrhea virus into host cells by interfering with clathrin-mediated endocytosis of the virus (27). If this is also true for SARS-CoV-2 entry into host cells, it is possible that the release of mortalin in COVID-19 patients into their blood, accompanied by reduced intracellular mortalin levels, makes the patients’ cells more exposed to SARS-CoV-2 virus entry. However, this is still open to investigation.

In conclusion, our results suggest that quantification of circulating mortalin and activated complement proteins in the blood of COVID-19 patients will enable an early identification of patients at risk of developing a severe disease. Besides being a good prognostic marker, it is possible that mortalin plays an important role either in disease progression and/or in disease control. If mortalin plays a significant role in disease control, it should be further investigated and considered as a therapeutic target for COVID-19 disease.

## Data availability statement

The original contributions presented in the study are included in the article/supplementary material. Further inquiries can be directed to the corresponding author.

## Ethics statement

The studies involving humans were approved by Hungarian Scientific and Research Ethics Committee (ETT-TUKEB; No. IV/4403–2/2020/EKU). The studies were conducted in accordance with the local legislation and institutional requirements. The participants provided their written informed consent to participate in this study.

## Author contributions

MA: Project administration, Writing – original draft, Data curation. GS: Data curation, Investigation, Methodology, Software, Writing – original draft, Writing – review & editing. LH: Investigation, Methodology, Software, Writing – review & editing. ZP: Conceptualization, Funding acquisition, Methodology, Resources, Supervision, Writing – review & editing. ZF: Conceptualization, Formal analysis, Funding acquisition, Methodology, Project administration, Resources, Supervision, Writing – original draft, Writing – review & editing.
